# Self-Assembling Peptide Hydrogels for Mucosal Drug Delivery: Synthesis, Triggering Strategies, and Therapeutic Applications

**DOI:** 10.3390/polym18212741

**Published:** 2026-11-04

**Authors:** Xin Zheng, Susu Jin, Shuyao Zhu, Tao Lee, Zhaohui Jin

**Affiliations:** Department of Pharmacy, West China Hospital, Sichuan University, Chengdu 610207, China

**Keywords:** self-assembling peptide hydrogels, mucosal drug delivery, controlled release

## Abstract

Self-assembling peptide hydrogels (SAPHs) have emerged as promising platforms for mucosal drug delivery due to their excellent biocompatibility, programmable sequence design, and diverse triggering strategies. This review systematically discusses the molecular basis, synthesis techniques, and triggering strategies of SAPHs, highlighting how these elements collectively enable in situ gelation and on-demand drug release at mucosal sites. We also review applications across the oral, gastrointestinal, ocular, vaginal, middle ear, and respiratory mucosa, demonstrating the therapeutic potential of SAPHs in infection treatment, tissue repair, and immunomodulation. We further compare SAPHs with other mucosal delivery systems and summarize the clinical status of mucosal delivery products; although several products are in clinical use, no SAPH-based product has been approved for mucosal delivery, and the factors limiting clinical translation are discussed. Despite significant progress, several challenges remain, including balancing mechanical stability with degradation, ensuring batch-to-batch consistency, confirming long-term biosafety, and obtaining regulatory approval. With the integration of artificial intelligence-assisted design and modular programmable platforms, SAPHs hold considerable promise for precision mucosal drug delivery.

## 1. Introduction

Mucosal tissues cover multiple anatomical sites, including the gastrointestinal tract, respiratory tract, reproductive tract, ocular surface, and middle ear, serving as critical barriers between the organism and the external environment [1,2,3]. Mucosal drug delivery offers several advantages, such as avoiding hepatic first-pass metabolism, enhancing bioavailability, and enabling localized targeted therapy [4,5]. However, it also faces formidable challenges, including the mucus barrier, enzymatic degradation, mucociliary clearance, and rapid dilution. Conventional topical formulations generally suffer from short mucosal retention time and poor drug permeability [6,7,8]. Therefore, the development of novel delivery systems capable of efficiently penetrating physiological barriers, achieving intelligent drug release, and possessing mucoadhesive properties has emerged as a critical scientific challenge urgently requiring resolution in the field [9,10].

Among the diverse materials explored for mucosal delivery, self-assembling peptide hydrogels (SAPHs) have emerged as a prominent research focus in recent years [11,12]. Peptides can spontaneously assemble into nanofibrous structures under specific conditions via non-covalent interactions and ultimately form three-dimensional hydrogel networks [13,14]. These materials not only possess excellent biocompatibility, biodegradability, and stability under physiological conditions but also allow for precise modulation of their mucoadhesive properties, mechanical performance, and responsiveness to mucosal microenvironmental signals through rational sequence design [15,16,17].

SAPHs offer several functional advantages for mucosal drug delivery. Their three-dimensional porous networks and abundant surface functional groups enable interactions with mucin in the mucus layer, thereby prolonging local retention [18,19]. Moreover, the incorporation of mucosal-penetrating motifs can enhance drug transport across mucosal barriers [20]. Furthermore, SAPHs can respond to changes in local environmental cues—including pH, metal ions, and solvent—facilitating controlled, sustained, and targeted drug release [21]. Collectively, these attributes position SAPHs as highly promising platforms for mucosal delivery across diverse anatomical sites.

The clinical translation of SAPHs for mucosal drug delivery remains at an early stage. To date, no SAPH-based mucosal delivery system has received regulatory approval for clinical use. Most studies are limited to preclinical animal models. This review therefore also discusses the barriers that have prevented clinical translation, including material, physiological, engineering, and regulatory challenges.

From an interdisciplinary perspective bridging materials science and pharmaceutics, this review summarizes the synthesis, trigger strategies, and recent advances in the application of SAPHs for mucosal drug delivery. It aims to provide a reference for future research and clinical translation in this field.

Before focusing on SAPHs, it is instructive to compare them with other hydrogel systems developed for mucosal drug delivery. Table 1 summarizes the building blocks, advantages, limitations, and mucosal applications of natural polymer hydrogels, synthetic polymer hydrogels, peptide–polymer hybrid hydrogels, hydrogel nano/microcapsules, microneedles, and hybrid systems. This comparison highlights the unique position of SAPHs—particularly their programmable sequence design and stimuli-responsive in situ gelation—while also underscoring shared challenges such as mechanical strength, batch consistency, and clinical translation.

## 2. Molecular Basis of SAPHs for Mucosal Drug Delivery

**Figure 1:**
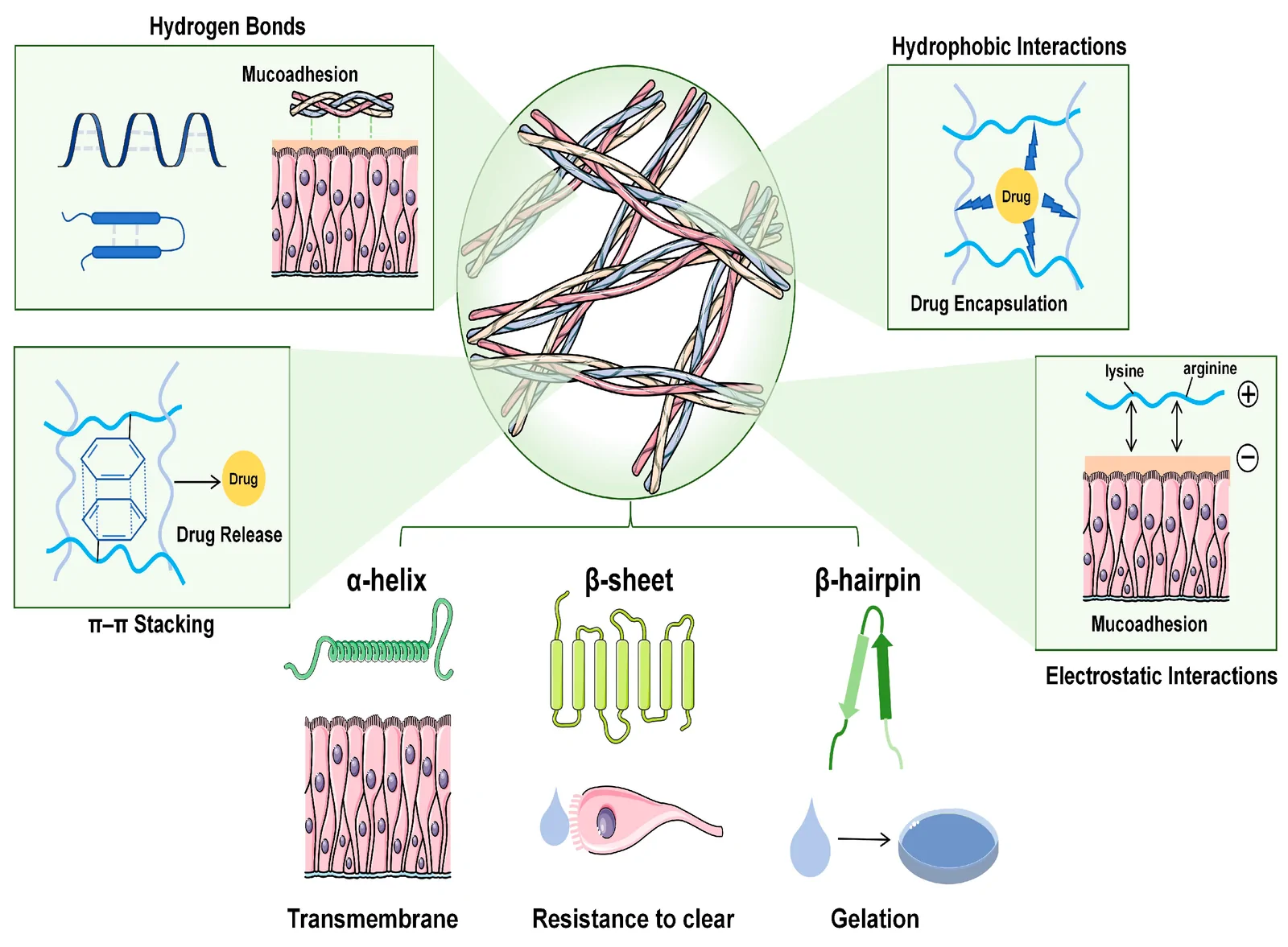
**From non-covalent interactions and secondary structures to mucosal delivery functions of SAPHs.**

The self-assembly of peptides into hydrogels is driven primarily by the synergistic effects of multiple non-covalent interactions (Figure 1) [22]. The reversibility and dynamic nature of these interactions endow the hydrogels with stimulus-responsiveness, self-healing capacity, and biodegradability, which confer distinct advantages in mucosal drug delivery [23,24].

### 2.1. Non-Covalent Interactions and Secondary Structures

Non-covalent interactions (hydrogen bonds, hydrophobic interactions, π–π stacking, and electrostatic interactions) stabilize secondary structures (α-helix, β-sheet, and β-hairpin), which in turn govern key mucosal delivery functions, including mucoadhesion, drug encapsulation and release, transmembrane penetration, resistance to clearance, and gelation.

Hydrogen bonding serves as the most fundamental interaction, stabilizing secondary structures such as α-helices and β-sheets [25,26]. It not only determines the mechanical stability and self-healing rate of the hydrogel but also mediates interactions between peptide molecules and mucin, representing an important molecular basis for mucoadhesion [27,28]. Hydrophobic interactions drive the aggregation of hydrophobic amino acid residues to form hydrophobic cores, which facilitate the encapsulation of hydrophobic drugs [29,30,31]. π–π stacking reinforces intermolecular aggregation through interactions between aromatic rings, resulting in a denser hydrogel network that contributes to sustained drug release [32,33]. Electrostatic interactions provide another key mechanism for mucoadhesion. Mucin on mucosal surfaces typically carries a negative charge at physiological pH. Therefore, designing peptides enriched with cationic amino acids (such as lysine and arginine) can exploit electrostatic attraction to strengthen hydrogel adhesion to the mucosa [34,35].

Structurally, the β-sheet is the most prevalent motif, conferring high mechanical strength to the hydrogel and enabling it to resist physical clearance mechanisms including gastrointestinal peristalsis, ciliary beating, and saliva or tear flushing [36,37,38,39]. The α-helix is commonly found in cell-penetrating peptides with transmembrane capability, facilitating the transport of loaded drugs across the mucosal epithelial barrier [40]. The β-hairpin, in turn, endows the hydrogel with “on-demand gelation” capability—existing as a low-viscosity solution for convenient administration, while rapidly transitioning into a gel upon reaching mucosal surfaces under physiological temperature or specific pH conditions, achieving in situ retention [41,42].

Collectively, these secondary structures endow SAPHs with distinct mechanical and responsive properties: β-sheets provide mechanical strength and resistance to clearance, α-helices facilitate transmembrane penetration, and β-hairpins enable on-demand gelation. Non-covalent interactions fine-tune these functions at the molecular level, regulating adhesion, encapsulation, and release behavior. This structure–property–function relationship provides a rational basis for designing SAPHs tailored to specific mucosal delivery requirements.

### 2.2. Thermodynamics and Kinetics of Self-Assembly

The self-assembly of peptides into hydrogels is governed by both thermodynamic and kinetic factors [43]. Thermodynamically, self-assembly is driven by a decrease in free energy, which arises from the balance between enthalpic gains (e.g., hydrogen bonding, electrostatic attraction, π–π stacking) and entropic changes (e.g., release of water molecules from hydration shells) [44,45]. The critical aggregation concentration (CAC) is a key parameter: below the CAC, peptides exist as monomers; above it, self-assembly proceeds spontaneously [46,47]. Cooperativity is also important—many SAPHs exhibit cooperative assembly, where initial nucleation is followed by rapid elongation [45,48]. Kinetic factors, such as nucleation rate, elongation rate, and the presence of kinetic traps, determine whether assembly leads to ordered nanofibers or amorphous aggregates [49,50].

Ionic strength, pH, and temperature strongly influence these processes [51,52]. For example, increasing ionic strength screens electrostatic repulsion, often promoting assembly of charged peptides [45,51]. pH changes alter the ionization state of amino acid side chains, affecting electrostatic interactions and hydrogen bonding [46,51]. Temperature affects hydrophobic interactions and hydrogen bonding, with higher temperatures (>60 °C) typically weakening these interactions [45,52,53]. Water molecules play a critical role: they mediate hydrogen bonding, participate in hydrophobic hydration, and influence the dynamic exchange of peptide molecules between solution and assembled states [54].

### 2.3. Interactions with Mucin and Mucus Layer

The interaction between SAPHs and the mucus layer is not limited to electrostatic attraction [55]. Hydrophobic interactions between hydrophobic peptide residues and hydrophobic domains of mucin can contribute to mucoadhesion [56,57]. Van der Waals forces provide additional weak interactions [57,58]. Mechanical anchoring occurs when hydrogel fibers interpenetrate the mucus network, increasing retention [59,60]. Polymer complexes can form between peptide chains and mucin glycoproteins, and electrostatic complexes or coacervates may form when oppositely charged species interact [61,62]. Counterion exchange dynamics also play a role: ions in mucus can exchange with counterions on the peptide, altering assembly and adhesion [63].

Quantitative characterization methods include zeta potential (surface charge), turbidimetry (assembly kinetics), confocal microscopy (mucus penetration), rheology (mechanical properties), and surface plasmon resonance (binding affinity) [64,65,66,67]. These methods should be applied to systematically compare SAPHs across different mucosal sites.

Therefore, the secondary structures of SAPHs govern their mechanical properties and response modes, while their non-covalent interactions regulate adhesion, encapsulation, and release behaviors at the molecular level [68,69]. Understanding how the molecular basis of SAPHs relates to their mucosal delivery functions is essential for designing efficient delivery systems [70].

Despite progress in understanding non-covalent interactions and mucin adhesion, several gaps remain. The thermodynamics and kinetics of self-assembly in complex mucosal fluids (containing mucin, salts, enzymes, and cells) are poorly understood. Predictive models linking sequence, assembly behavior, and mucosal performance are lacking. Future studies should systematically quantify CAC, cooperativity, and kinetic pathways under physiologically relevant conditions, and develop standardized methods for comparing mucin interactions across SAPH systems.

## 3. Synthesis of SAPHs for Mucosal Drug Delivery

The synthesis of peptides is the first step in preparing SAPHs, and the quality of the synthesized peptides directly determines the reproducibility of subsequent self-assembly behaviors and the stability of hydrogel performance [71]. Currently, peptide synthesis relies primarily on solid-phase peptide synthesis (SPPS) [72].

### 3.1. Solid-Phase Peptide Synthesis

SPPS is the most mature and widely used technology for preparing self-assembling peptides [72]. Its core principle involves covalently attaching the C-terminal amino acid of the target peptide chain to an insoluble resin support, followed by iterative cycles of coupling-washing-deprotection-washing to sequentially extend the peptide chain from the C-terminus to the N-terminus [73,74]. In the preparation of self-assembling peptides, the most commonly employed protection strategy is the Fmoc/tBu strategy—Fmoc (9-fluorenylmethoxycarbonyl) is used to protect the α-amino group, which can be selectively removed under mild basic conditions (e.g., 20% piperidine), while side-chain functional groups are protected with acid-labile groups such as tert-butyl [75]. Upon completion of peptide chain assembly, treatment with trifluoroacetic acid simultaneously achieves cleavage of the peptide from the resin and removal of side-chain protecting groups [76]. The application of automated peptide synthesizers enables high-throughput, high-reproducibility large-scale production of peptides [77,78].

After synthesis, the peptides must be purified by high-performance liquid chromatography and their molecular weights confirmed by mass spectrometry [79,80,81]. High purity is essential for obtaining hydrogels with consistent batch-to-batch performance and reproducible properties. This is particularly critical for mucosal drug delivery studies, because trace impurities can alter self-assembly pathways and consequently affect mucoadhesion and drug release behavior [82].

### 3.2. Mucosal Drug Delivery-Oriented Functional Modifications

Researchers can introduce specific functional groups or bioactive sequences at the N- or C-terminus of the peptide. This step is key to endowing the hydrogel with mucosal delivery functions [83].

#### 3.2.1. Enhancing Mucosal Retention

Mucosal surfaces are continuously cleared by physical mechanisms such as ciliary beating, mucus flow, and tear flushing. Prolonging formulation retention on mucosal surfaces is a primary prerequisite for improving drug bioavailability. The incorporation of cationic residues such as lysine and arginine into the peptide sequence enables electrostatic attraction between the positive charges and the negatively charged mucin in the mucus layer, thereby enhancing the mucoadhesive force of the hydrogel [11]. N-terminal Fmoc modification represents another effective strategy. The bulky aromatic structure of the Fmoc group not only promotes peptide self-assembly through strong π–π stacking interactions but also further reinforces mucoadhesion by interacting with aromatic residues in mucin [84].

#### 3.2.2. Facilitating Transmembrane Delivery

The mucosal epithelial barrier is a major obstacle to the absorption of macromolecular drugs such as proteins and nucleic acids. Introducing cell-penetrating peptide sequences such as TAT and Penetratin into the peptide chain can transiently disrupt the integrity of epithelial cell membranes, facilitating drug transport across the mucosal barrier [85,86,87].

#### 3.2.3. Microenvironment-Responsive Release

Different mucosal sites possess characteristic physiological microenvironments—including the strongly acidic environment of the stomach, the near-neutral pH of the intestine, and the weakly acidic vagina [88,89]. By incorporating amino acids with ionizable groups, such as glutamic acid, pH shifts in the mucosal microenvironment can trigger sol–gel transitions or drug release [90,91]. Furthermore, embedding peptide segments that can be specifically cleaved by mucosa-associated enzymes, such as matrix metalloproteinases, enables enzyme-triggered in situ drug release, further enhancing the spatiotemporal precision of delivery [92].

#### 3.2.4. Improving Enzymatic Stability

The mucosal environment is rich in proteases, including trypsin and chymotrypsin in the gastrointestinal tract and lysozyme in the nasal cavity, rendering unmodified natural peptides susceptible to rapid degradation and inactivation [93,94,95]. Substituting natural amino acids with non-natural amino acids or D-amino acids in the sequence effectively resists protease recognition and degradation, thereby prolonging the effective duration in the mucosa [96,97]. However, this increased stability comes at a cost, as increasing the number of D-amino acid substitutions has been shown to be accompanied by increased cytotoxicity in cell culture [98].

### 3.3. Design of Self-Assembling Sequences

The design of self-assembling peptide sequences follows several general principles. β-sheet-forming sequences often rely on alternating hydrophobic and hydrophilic residues (e.g., (RADA)_4_, (FKFE)_2_) [99,100]. α-helical sequences typically contain repeating heptad patterns (e.g., coiled-coil motifs) [101]. β-hairpin sequences require a turn motif (e.g., VPGVG) flanked by β-strand segments [102]. Amphiphilic peptides with a hydrophobic tail and a hydrophilic head can self-assemble into nanofibers [103,104]. Aromatic residues (Phe, Tyr, Trp) promote π–π stacking and often enhance mechanical strength [105,106]. Cationic residues (Lys, Arg) can improve mucoadhesion, while anionic residues (Glu, Asp) enable pH responsiveness [51,107,108]. Enzyme-cleavable sequences (e.g., MMP-2 substrates) allow pathological microenvironment-triggered release [109]. Cell-penetrating peptides (TAT, Penetratin) facilitate transmembrane delivery [85]. Non-natural and D-amino acids improve enzymatic stability but may increase cytotoxicity [98]. A summary of representative sequences and their functions is provided in Table 2.

### 3.4. Drug Release Mechanisms and Stability

Drug release from SAPHs is governed by several mechanisms: diffusion through the hydrogel network, erosion or degradation of the network, swelling-controlled release, and stimuli-triggered release (e.g., pH, enzymes, redox) [110,111]. The release profile depends on mesh size, crosslinking density, drug–peptide interactions, and degradation rate [112,113,114,115]. Hydrophobic drugs are typically encapsulated in hydrophobic cores and released slowly, while hydrophilic drugs diffuse rapidly [116]. Macromolecular drugs (proteins, nucleic acids) may interact electrostatically with the peptide network, modulating release [110,117,118].

Stability of loaded substances can be affected by the hydrogel environment [119,120]. For example, acidic or enzymatic conditions may degrade sensitive drugs. Strategies to mitigate this include encapsulation within hydrophobic pockets, covalent conjugation, and use of non-natural amino acids to reduce protease access [116,121,122]. Functional properties of drugs may change upon interaction with peptides, including altered solubility, stability, and activity [123]. These interactions should be characterized using circular dichroism, fluorescence spectroscopy, and mass spectrometry [124,125,126].

### 3.5. Other Peptide-Based Hydrogel Architectures for Mucosal Delivery

Beyond conventional bulk hydrogels, peptide-based materials have been engineered into alternative architectures for mucosal drug delivery. Peptide hydrogel nano- and microcapsules offer high encapsulation efficiency and controlled release, but often suffer from limited mucosal retention [61,127]. Peptide-based microneedles can bypass the mucus barrier and deliver drugs directly into mucosal tissue, though their application is restricted to accessible sites such as the buccal and vaginal mucosa [128,129]. Hybrid systems combining peptides with liposomes, nanoparticles, or synthetic polymers integrate the programmability of peptides with the mechanical robustness or loading capacity of other materials [85,130,131]. These architectures expand the design space of peptide-based mucosal delivery but introduce additional challenges in manufacturing consistency and regulatory classification.

While SPPS is well established, the design of self-assembling sequences for mucosal delivery remains largely empirical. Systematic structure–function relationships linking sequence motifs to mucoadhesion, penetration, and release are lacking. Drug release mechanisms are often inferred rather than directly measured. Future work should integrate computational design, high-throughput synthesis, and standardized release testing to accelerate the discovery of optimized SAPH sequences.

## 4. Triggering Strategies for SAPHs Applied to Mucosal Drug Delivery

**Figure 2:**
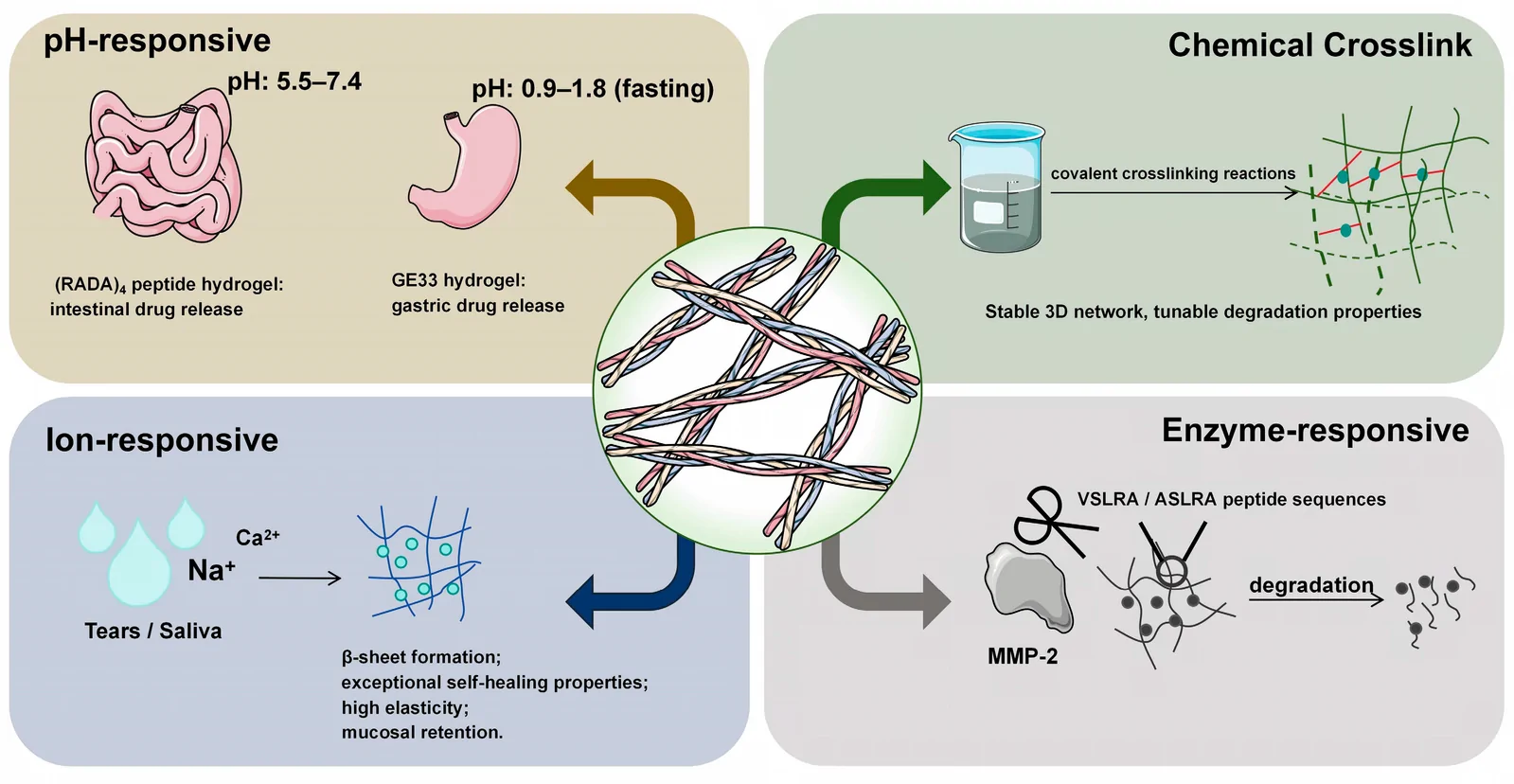
**Triggering strategies for SAPHs in mucosal drug delivery.** SAPHs can be engineered to respond to mucosal microenvironmental cues via pH-responsive (gastric vs. intestinal release), ion-responsive (Na^+^/Ca^2+^ in tears/saliva), chemical crosslinking, and enzyme-responsive (MMP-2 cleavage) mechanisms, enabling precise spatiotemporal control over gelation, degradation, and drug release.

In mucosal drug delivery applications, the selection of triggering strategies is particularly critical—not only because it determines the microstructure and mechanical properties of the hydrogel, but also because it directly affects the in situ gelation capacity of the formulation on mucosal surfaces, its compatibility with mucosal tissues, and drug release behavior [132,133,134]. Therefore, appropriate triggering strategies are needed to direct the self-assembly or self-degradation of SAPHs under different physiological conditions and requirements (Figure 2) [135].

### 4.1. Physical Induction Methods

Physical induction methods trigger self-assembly by altering external physical conditions such as temperature, pH, ionic strength and light intensity [131]. These approaches are simple, mild, and free of additional chemical reagents, making them the most commonly used route for preparing SAPHs [133,136]. For mucosal drug delivery, a significant advantage of physical induction methods lies in their capacity for in situ gelation—the formulation remains a low-viscosity solution ex vivo, facilitating spray, instillation, or injection administration; upon reaching the mucosal surface, it rapidly forms a gel in response to the local pH or ionic environment, achieving close adhesion and prolonged retention [16,137].

pH-responsive transformation of SAPHs exploits the characteristic pH of different mucosal sites, enabling site-specific efficacy [16,138]. For example, the antimicrobial peptide GE33 hydrogels can self-assemble under neutral pH conditions and disassemble in the acidic gastric environment to achieve targeted killing of Helicobacter pylori [139]. Furthermore, N-terminal appending residue modifications not only modulate the self-assembly of the (RADA)_4_ peptide but also enable intestinal-specific release of encapsulated drugs [16].

The pH values along the gastrointestinal tract vary with physiological state [140]. The fasting gastric pH is approximately 0.9–1.8, but it can rise to 3.5–5.0 after food intake [141,142]. The intestinal pH is approximately 5.5–7.4, with the colon around 6.0–7.0 [143]. These values should be considered when designing pH-responsive SAPHs.

Ion-induced gelation uses electrolytes naturally present in mucosal fluids as triggers. Changes in ionic strength or charge screening modulate electrostatic interactions and drive peptide assembly [137,144]. This strategy holds particular value in ocular and oral mucosal delivery, where tears and saliva are abundant. The charged peptide Z5 exhibits Na+-triggered self-assembly into a nanofiber network with high β-sheet content, and its hydrogel in normal saline shows exceptional self-healing properties [145]. Furthermore, calcium ions naturally present in tear fluid can enhance the elasticity of SAPHs, thereby prolonging pre-corneal drug retention [123].

Temperature-responsive SAPHs have been developed for mucosal delivery [146,147]. For example, poly(N-isopropylacrylamide)-peptide conjugates undergo sol–gel transition near physiological temperature, enabling in situ gelation on mucosal surfaces [148,149]. Elastin-like polypeptides (ELPs) also exhibit temperature-dependent self-assembly and have been used for mucosal drug delivery [150,151,152].

Light-responsive SAPHs incorporate azobenzene or spiropyran moieties to enable light-triggered assembly/disassembly [153]. Azobenzene undergoes reversible trans–cis isomerization under UV–vis light, disrupting π–π stacking and altering nanostructure [154,155,156]. Visible-light-driven transient nanofiber hydrogels from peptide-spiropyran conjugates have also been developed [157]. These systems offer spatiotemporal control for ocular or oral delivery, though mucosal studies remain scarce.

### 4.2. Chemical and Enzymatic Triggering Strategies

Beyond physical induction, chemical and enzymatic strategies provide versatile routes to trigger SAPH assembly, crosslinking, degradation, and drug release. Chemical crosslinking—via Michael addition, Schiff base formation, disulfide bonding, or enzymatic crosslinking—yields stable three-dimensional networks with tunable mechanical strength and degradation [158]. This approach is well suited for mucosal delivery scenarios that require long-term retention and sustained drug release [159]. These chemistries can further be designed to respond to pathological cues such as glutathione, glucose, and reactive oxygen species, while enzyme-catalyzed precursor conversion enables MMP-triggered in situ gelation. Collectively, chemical and enzymatic triggering strategies extend the trigger–assembly–function paradigm of SAPHs for mucosal drug delivery.

Redox-responsive SAPHs exploit elevated glutathione (GSH) in inflamed or tumor tissues to cleave disulfide bonds, triggering drug release [61,160]. Hybrid nanogels crosslinked with cystine disulfide bonds exhibited GSH-responsive release and enhanced mucus penetration [61]. Additionally, a peptide metallohydrogel showed rapid reduction by ascorbate with a 160-fold stiffness change [161]. This redox-triggered strategy enables site-specific drug release in inflammatory mucosal diseases [160,162].

Glucose-responsive SAPHs use boronic acid–diol interactions to sense glucose and trigger insulin release [163,164]. Phenylboronic acid (PBA) reversibly binds glucose, forming or cleaving boronic ester crosslinks [165]. Glucose-responsive insulin delivery systems based on pH-responsive peptide hydrogels and glucose oxidase have been reported [162,166]. This strategy is relevant for oral or buccal delivery, but SAPH-specific studies remain limited, as most systems are polymer-based [165].

ROS-responsive SAPHs employ thioketal or phenylboronic ester linkages cleaved by reactive oxygen species (ROS), which are elevated in inflammatory mucosal diseases [167,168]. Thioketal nanoparticles released siRNA orally delivered to intestinal inflammation sites [169]. ROS-cleavable peptide sequences have also been used to graft hydrogels with ROS responsiveness [168]. This strategy is promising for mucosal diseases such as IBD and periodontitis [169,170].

Enzyme-catalyzed transformation of SAPHs uses specific enzymes to catalyze the conversion of water-soluble peptide precursors (e.g., dephosphorylation, hydrolysis) to generate peptide gelators with enhanced self-assembly propensity, triggering self-assembly under physiological conditions [171,172,173]. This strategy offers unique advantages for mucosal delivery. Inflamed mucosal sites often display markedly elevated levels of matrix metalloproteinases (MMPs), which can be exploited to achieve pathological microenvironment-triggered in situ gelation and on-demand drug release [174,175,176]. Novel self-assembled peptide hydrogels (EDP1 and EDP2) have been developed, which incorporate MMP-2-cleavable peptide sequences (VSLRA and ASLRA), self-assemble into nanofibrous networks, and exhibit enzyme-responsive degradation upon exposure to MMP-2 [176].

The unique feature of SAPHs lies in their integrated molecular mechanism of trigger-assembly-function [135]. The peptides, under triggering conditions, undergo synergistic reconstruction through non-covalent interactions to form nanofibrous networks with well-defined secondary structures [52]. Moreover, rational sequence design enables biological functional modules—such as mucoadhesive segments, enzyme-cleavable sites, or cell-penetrating peptides—to be directly incorporated into the gel scaffold [177]. Therefore, triggering strategies in peptide systems represent not merely a physical phase transition, but rather a spatiotemporal switch for biological functional activation [134,162].

Physical and chemical triggering strategies have been extensively explored in vitro, but their performance in dynamic mucosal environments remains poorly characterized. Most studies use static conditions that do not recapitulate mucus flow, pH fluctuations, or enzymatic gradients. Multi-stimuli-responsive systems are promising but complex to manufacture. Future studies should validate triggering strategies in physiologically relevant ex vivo and in vivo models.

## 5. Applications of SAPHs in Mucosal Drug Delivery

**Figure 3:**
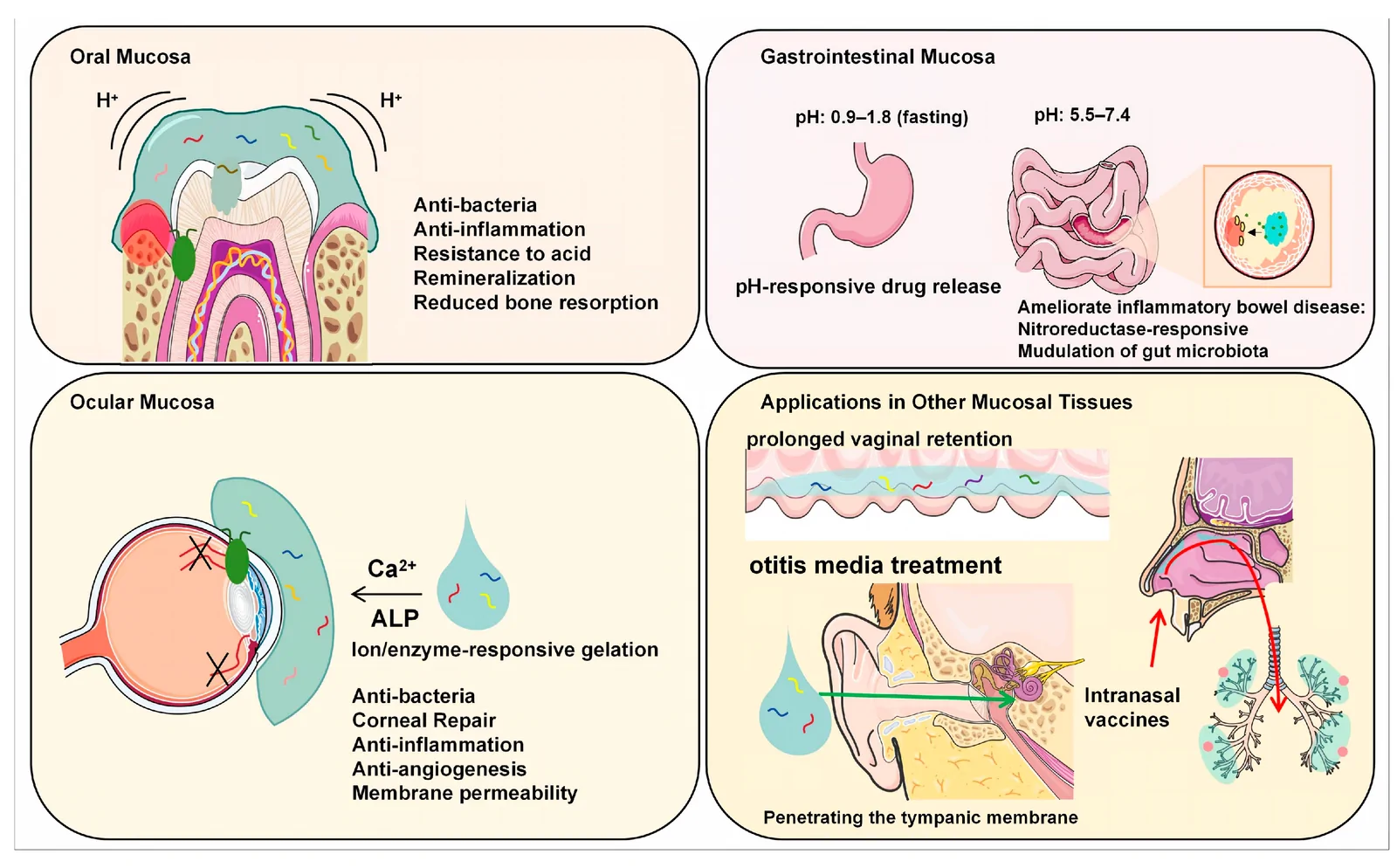
**Applications of SAPHs across mucosal sites.** SAPHs have been tailored for distinct mucosal environments—oral (anti-bacterial, anti-inflammatory, remineralization), ocular (ion/enzyme-responsive gelation, corneal repair), gastrointestinal (pH-responsive release, microbiota modulation), vaginal (prolonged retention), middle ear (tympanic membrane penetration), and respiratory (intranasal vaccination)—achieving targeted and stimuli-responsive drug delivery.

SAPHs have been investigated across a broad spectrum of mucosal sites, including the oral and gastrointestinal tract, the ocular surface, and the vaginal, middle ear, and respiratory mucosa (Figure 3). By leveraging programmable sequence design and diverse triggering mechanisms, these materials can overcome site-specific physiological barriers to achieve targeted, sustained, or stimuli-responsive drug delivery. The following sections review these applications, highlighting how SAPHs address the distinct challenges posed by each mucosal environment. Representative studies of SAPHs applied in various mucosal sites are summarized in Table 3.

### 5.1. Oral and Gastrointestinal Mucosal Delivery

The oral and gastrointestinal mucosa, spanning from the oral cavity to the gastrointestinal tract, represents an important route of drug administration [178]. However, this route faces formidable physiological barriers: continuous salivary flushing and lysozyme degradation in the oral cavity, the highly acidic gastric environment and pepsin digestion in the stomach, and the combined effects of digestive enzymes and the mucus barrier in the intestine, all of which collectively impede drug absorption [179]. Moreover, the marked pH gradient along the digestive tract—near-neutral in the oral cavity, pH 0.9–1.8 (fasting) in the stomach, and pH 5.5–7.4 in the intestine—requires delivery systems to achieve site-specific release [16,180]. SAPHs offer programmable sequence design, diverse triggered assembly mechanisms, and excellent biocompatibility. Through rational sequence engineering, they can overcome these challenges and enable targeted, controlled release [52,181].

#### 5.1.1. Oral Disease Treatment

Dental caries and periodontitis represent the two most common chronic infectious diseases in dentistry, and SAPHs have been explored in both directions. A key challenge in caries management is that conventional antimicrobials do not distinguish between pathogenic and commensal microbiota. Long-term use disrupts the oral microbial ecosystem, and antimicrobial action alone cannot restore demineralized dental hard tissues [182,183,184,185]. The multifunctional self-assembling peptide 8DSS-C8-P-113 was designed to address this challenge. This molecule integrates three functional domains—the antimicrobial peptide P-113, the adhesion-promoting peptide C8, and the remineralization-promoting domain 8DSS—into a single entity. Upon self-assembly into a hydrogel, it adheres stably to the tooth surface, resists acid attack, exerts antibiofilm activity against Streptococcus mutans, and induces enamel remineralization to restore demineralized hard tissues [186]. The therapeutic rationale for periodontitis differs fundamentally, centering on controlling local inflammation and inhibiting alveolar bone resorption [187,188,189]. The PA/GL13K system operates on a charge-complementary principle—negatively charged peptide amphiphiles (PA) electrostatically associate with the positively charged antimicrobial peptide GL13K, forming a hydrogel that enables sustained release of the antimicrobial peptide [190]. In a mouse model of periodontitis, local injection of this hydrogel attenuated inflammatory infiltration and reduced osteoclast-mediated bone resorption, effectively mitigating periodontal tissue destruction [190]. Collectively, these findings indicate that SAPHs in oral disease management not only serve as passive drug carriers but also actively participate in tissue repair.

Oral SAPHs have demonstrated promising antibacterial, anti-inflammatory, and remineralization effects in preclinical models. However, most studies are limited to small animal models, and long-term safety and efficacy in humans remain unknown. Future studies should focus on large animal models, standardized evaluation of mucoadhesion and drug release, and development of multi-functional SAPHs that simultaneously address infection, inflammation, and tissue repair.

#### 5.1.2. Gastrointestinal Disease Treatment

Beyond the oral cavity, SAPHs have shown considerable promise in gastrointestinal drug delivery. In gastric mucosal injury, the rapidly assembling Rqdl10 peptide hydrogels effectively encapsulate TFF3 peptides, protecting them from rapid degradation by gastric acid and pepsin. They then promote gastric mucosal repair through sustained release while exerting anti-inflammatory effects [11]. When intestinal targeted delivery is required, N-terminally modified acid-stable G(RADA)_4_ peptide hydrogels exhibit drug release of less than 5% in gastric acid but trigger over 70% release in the gut, achieving pH-responsive intestinal targeting [16]. Furthermore, nitroreductase-responsive peptide hydrogels can encapsulate live cells such as *Escherichia coli*, shielding them from gastric acid and bile salts during oral administration. Upon reaching the intestine, the upregulated nitroreductase specifically degrades the hydrogel, releasing live cells to modulate the gut microbiota and ameliorate inflammatory bowel disease [191]. These studies demonstrate that SAPHs not only possess inherent designability but also adapt their responsive behavior to the distinct microenvironmental characteristics of different gastrointestinal segments, providing a versatile platform for oral drug and live-cell delivery.

Gastrointestinal SAPHs have achieved pH-responsive release and live-cell delivery in preclinical models. However, the complex and dynamic nature of the gastrointestinal tract—including food intake, microbiota, and peristalsis—poses challenges for predictable drug release. Future work should incorporate real-time monitoring, patient-specific pH profiles, and long-term safety assessment.

### 5.2. Ocular Mucosal Delivery

Ocular mucosal drug delivery faces multiple challenges, including tear flushing, the corneal barrier, and ocular surface clearance. Conventional eye drops suffer from short precorneal retention time and low bioavailability, often necessitating frequent and high-dose administration, which increases the risk of ocular surface irritation and enzymatic inhibition [192]. Rationally designed SAPHs improve ocular drug bioavailability through tunable assembly behavior and shear-responsive morphological transitions [193].

#### 5.2.1. Ocular Infectious Disease

Bacterial keratitis is a prevalent ocular infection that, if not treated promptly, can progress to corneal ulceration, perforation, and even blindness [194,195]. In this condition, conventional antibiotic eye drops face the additional barrier of poor corneal penetration. This limitation, combined with the inherently short precorneal retention of eye drops, necessitates frequent high-dose administration, which in turn raises the risk of ocular surface irritation and enzymatic inhibition [123,196,197]. Current strategies covalently conjugate antibiotics to peptides, creating self-assembling conjugates that form supramolecular hydrogel eye drops with significantly prolonged precorneal retention [123]. Pan et al. developed a calcium ion-responsive chloramphenicol–peptide conjugate that self-assembles into a supramolecular hydrogel. This hydrogel extends the precorneal retention of the antibiotic, exhibits no toxicity to normal corneal epithelial cells, reduces ocular irritation, and demonstrates favorable therapeutic outcomes. This biomimetic strategy achieves an integrated “drug-as-carrier” approach, offering a new paradigm for ocular anti-infective therapy [123].

#### 5.2.2. Corneal Repair

The quality of corneal repair following injury directly affects visual function, and delayed corneal epithelial healing can lead to infection, corneal opacification, and even perforation [198,199,200]. SAPHs can incorporate corneal repair-active peptide sequences, functioning as “drug-as-carrier” systems that promote epithelial cell migration and regeneration [193,201]. For example, the Nap-FFPHSRN hydrogels, generated by covalent conjugation of a self-assembling motif (Nap-FFF) with the fibronectin-mimetic peptide sequence PHSRN, enhance the proteolytic stability of PHSRN, increase cell migration, and strengthen corneal epithelial tight junctions through upregulation of zonula occludens-1 (ZO-1) expression, achieving complete restoration of corneal morphology and structure [202]. The Nap-YpYY-TB500 hydrogels exploit the elevated alkaline phosphatase (ALP) activity at ocular surface injury sites to achieve enzyme-responsive in situ self-assembly, endowing the hydrogel with spatiotemporally controllable corneal repair capability [203].

Corneal wound healing is a complex and dynamic process that involves not only epithelial cell migration and proliferation but also concurrent inflammatory responses and neovascularization [204,205]. Inflammation initiates tissue repair, but excessive or persistent inflammation exacerbates damage and delays healing [206,207]. Meanwhile, although neovascularization may provide nutrients to injured tissue, it disrupts the normal corneal microarchitecture, causing visual impairment or even blindness [208,209]. Therefore, an ideal corneal repair strategy should promote epithelial regeneration while suppressing excessive inflammation and pathological neovascularization. SAPHs, through covalent conjugation of anti-inflammatory or anti-angiogenic agents to self-assembling peptides, have shown significant therapeutic potential in this regard [210,211]. Chen et al. covalently conjugated two dexamethasone molecules to a short peptide (Tyr-Glu-Asn-Pro-Thr-Tyr) to create a self-assembling anti-inflammatory supramolecular hydrogel eye drop [210]. This hydrogel undergoes gel–sol–gel transitions in response to shear force, thereby increasing precorneal retention time [210]. In a rodent model of uveitis, this hydrogel eye drop effectively suppressed inflammation without causing ocular irritation [210]. The aflibercept-loaded hydrogel formulation (Gel 2_1&Eylea), utilizing cell-penetrating peptide 1 as a delivery vehicle, exhibited excellent membrane permeability and significantly reduced neovascular density in a rabbit corneal neovascularization model, without adverse effects on normal limbal vessels [211]. Collectively, these findings show that SAPHs in ocular delivery have evolved from simple drug carriers to intelligent platforms that integrate targeted delivery, microenvironmental responsiveness, and biological modulation.

Ocular SAPHs have shown efficacy in corneal repair and anti-infection. However, long-term safety data are lacking, and the impact of tear fluid composition on hydrogel stability needs further study. Future directions include multi-stimuli-responsive systems (pH/enzyme/ROS) and standardized evaluation of corneal repair.

### 5.3. Applications in Other Mucosal Tissues

Beyond the gastrointestinal tract, oral cavity, and ocular surface, SAPHs have also demonstrated unique advantages in drug delivery to the vaginal, middle ear, and respiratory mucosa [212,213,214].

#### 5.3.1. Vaginal Mucosal Delivery

Vaginal drug delivery offers local targeting, avoidance of hepatic first-pass metabolism, and suitability for both local and systemic administration. However, the vagina is a dynamic physiological environment whose self-cleansing mechanisms pose substantial challenges [215]. Continuously secreted vaginal mucus, containing mucins, immunoglobulins, and various enzymes, forms a natural defensive barrier [216]. While protective, this barrier rapidly clears drug particles through mucus flow and shedding, significantly shortening their retention time [216]. Moreover, cyclical changes in mucus thickness during the menstrual cycle and dynamic fluctuations in the vaginal microbiota affect drug dissolution, release, and absorption, limiting the efficacy of conventional gels, creams, and suppositories [217]. Consequently, the development of novel delivery systems capable of prolonging vaginal retention time and achieving sustained drug release has become a research priority in the field of vaginal drug delivery [218]. Li et al. developed a composite system, PA-particles-TH, in which a Periplaneta americana extract was loaded into self-assembled spider silk protein particles and then embedded in a thermosensitive hydrogel (TH) [212]. This system exhibited excellent biocompatibility, achieved 24-h sustained drug release, and significantly prolonged vaginal retention time [212]. This study demonstrates the potential of combining self-assembled proteins with thermosensitive hydrogels for mucosal drug delivery, expanding the application from single-component hydrogels to multi-component integrated systems.

#### 5.3.2. Middle Ear Delivery

The central challenge in middle ear drug delivery is that drugs must penetrate the tympanic membrane to reach the middle ear cavity [219]. With a thickness of 30 to 230 μm, the tympanic membrane possesses a multi-layered keratinized epithelial structure and dense intercellular junctions that confer extremely low permeability, naturally impeding effective penetration of topically applied drugs [220,221,222]. Therefore, the membrane permeability of the delivery vehicle is a critical determinant of trans-tympanic drug delivery efficiency. Patel and colleagues proposed a novel strategy using dynamic SAPHs containing cell-penetrating peptides [213]. The hydrogels exploit their inherent capacity to transition from a one-dimensional nanofibrous network to zero-dimensional micelles, dissociating to generate an effective cell-penetrating peptide while achieving temporally controlled release of the antibiotic ciprofloxacin [213]. This approach opens new avenues for non-invasive local treatment of otitis media.

#### 5.3.3. Inhalation Immunization

Intranasal vaccines can induce mucosal immune responses, providing protection at the early stages of respiratory pathogen invasion, replication, and transmission [223,224,225]. However, the nasal mucus barrier, rapid mucociliary clearance, and epithelial tight junctions significantly limit vaccine uptake, while antigens administered intranasally often face the dual challenges of low bioavailability and weak immunogenicity [226,227,228,229]. To overcome these challenges, Fu and colleagues reported an injectable self-assembling hydrogel vaccine (RAgel-RBD vaccine) for protective immunity against respiratory viruses [214]. This highly bioadhesive hydrogel vaccine enhances antigen stability and prolongs retention in the nasal cavity and lungs by restricting the antigen to the mucosal surface [214]. This innovative intranasal vaccine formulation holds transformative potential for mucosal immunization against respiratory infectious diseases and cancers.

Vaginal, middle ear, and respiratory SAPHs remain in early preclinical stages. For vaginal delivery, the influence of the menstrual cycle on mucus properties must be considered. For middle ear delivery, tympanic membrane penetration efficiency needs improvement. For intranasal vaccines, mucosal immunogenicity should be enhanced. Future studies should develop SAPHs with stronger mucoadhesion and lower immunogenicity.

### 5.4. Comparison with Other Mucosal Delivery Systems

Table 4 compares SAPHs with other mucosal delivery systems, including nanoparticles, liposomes, micelles, hydrogels from natural polymers, and microneedles. SAPHs offer advantages in programmability, biocompatibility, and in situ gelation, but face challenges in mechanical strength, batch consistency, and clinical translation.

### 5.5. Clinical Status of Mucosal Delivery Products

Table 5 shows that several mucosal delivery products are already in clinical use, including nasal sprays (e.g., calcitonin), buccal tablets (e.g., fentanyl), ocular inserts (e.g., pilocarpine), and vaginal rings (e.g., contraceptives) [230]. However, no SAPH-based product has been approved for mucosal delivery. The main barriers include lack of standardized manufacturing, insufficient long-term safety data, and unclear regulatory pathways [168,231,232]. SAPHs differ from conventional products by offering programmable responsiveness and in situ gelation, but these advantages have not yet been translated into clinical benefit.

### 5.6. Cross-Mucosal Comparison of SAPH Design and Performance

The mucosal sites discussed above differ markedly in barrier properties, clearance mechanisms, and microenvironmental conditions. These differences demand site-specific design of SAPHs, and comparing them side by side clarifies how peptide sequence, self-assembly behavior, and hydrogel properties translate into delivery outcomes. Table 6 summarizes representative SAPH systems across mucosal sites and compares their design strategies, drug release mechanisms, mucosal retention, and penetration enhancement.

### 5.7. Summary of Applications

Across the mucosal sites reviewed above, SAPHs have demonstrated a consistent ability to overcome site-specific barriers through rational sequence design and microenvironment-responsive assembly. In the oral cavity and gastrointestinal tract, SAPHs exploit pH gradients and enzymatic cues to achieve site-specific release and tissue repair. On the ocular surface, ion- and enzyme-responsive gelation prolongs precorneal retention and supports corneal regeneration. In the vaginal, middle ear, and respiratory mucosae, SAPHs address rapid clearance, tympanic membrane impermeability, and weak mucosal immunogenicity, respectively. Table 6 further highlights that no single SAPH design is universally optimal: mucoadhesion is best achieved through cationic or aromatic modifications, release kinetics are dictated by local pH and enzyme profiles, and penetration enhancement relies largely on cell-penetrating peptides. These comparisons underscore that future SAPH development should follow a “site-specific design” principle, matching sequence, trigger, and mechanical properties to the anatomical and pathological features of each mucosal environment.

## 6. Challenges and Future Perspectives

Despite the significant progress of SAPHs in mucosal drug delivery, their clinical translation remains fraught with challenges. At the material level, the main technical bottleneck is the delicate balance between mechanical strength and degradation behavior [52,233]. Most hydrogels lack sufficient mechanical integrity to withstand the stresses encountered in the oral cavity and gastrointestinal tract. Extending peptide sequences or incorporating non-natural amino acids can improve stability, but these approaches may cause excessively slow degradation, foreign body accumulation, and impaired tissue remodeling [234,235]. In addition, batch-to-batch variability, sterilization effects on self-assembly, and storage stability need to be addressed.

At the physiological level, individual variability and the dynamic nature of the mucosal microenvironment (e.g., shifts in pH, enzyme profiles, and redox potential under inflammation) mean that current “static designs” optimized in animal models often fail to adapt to “dynamic” pathological conditions seen in patients [168]. Systematic evaluation of these dynamic factors remains insufficient. At the engineering level, scale-up production faces substantial challenges in batch-to-batch consistency, as self-assembly pathways are highly sensitive to parameters including peptide purity, temperature, and shear forces, and standardized quality control systems are lacking [168,236]. Process analytical technology (PAT) and quality-by-design (QbD) approaches are needed.

Furthermore, introducing non-natural amino acids or exogenous functional sequences may pose immunogenicity risks. The long-term biosafety and metabolic fate of degradation products also remain poorly characterized [51]. Regulatory hurdles, including ambiguous product classification and a lack of internationally harmonized standards, further impede clinical translation [237].

The development of SAPHs for mucosal drug delivery is likely to focus on several key directions. Artificial intelligence- assisted rational design can accelerate the discovery and optimization of novel peptide sequences by building predictive models that link peptide sequence, assembly behavior, and mucosal delivery performance [238,239]. Programmable design will enable the on-demand combination of functional modules—such as mucoadhesion, drug loading, transmembrane penetration, and immunomodulation—allowing “plug-and-play” customizable delivery systems [240,241]. Additionally, the establishment of standardized production processes and systematic evaluation of long-term biosafety will be essential steps toward clinical translation [242,243]. Organoid models for mucosal delivery evaluation and microfluidic technologies for high-throughput screening are also promising [244].

With the deepening integration of materials science, computational biology, pharmaceutics, and clinical medicine, SAPHs are poised to become important tools in the field of precision mucosal drug delivery.

## 7. Conclusions

SAPHs are three-dimensional networks formed by peptides through non-covalent interactions. Their excellent biocompatibility, programmable sequence design, diverse triggering mechanisms, and ability to adapt to the mucosal microenvironment confer unique advantages for mucosal drug delivery. This review has systematically examined the molecular basis, synthesis techniques, triggering strategies, and therapeutic applications of SAPHs. We have also compared SAPHs with other mucosal delivery systems, summarized the current clinical status of mucosal delivery products, and highlighted that no SAPH-based product has yet been approved for mucosal delivery. Despite encouraging preclinical results across oral, gastrointestinal, ocular, vaginal, middle ear, and respiratory mucosa, several challenges must be addressed before clinical translation. These include balancing mechanical stability with degradation, ensuring batch-to-batch consistency, confirming long-term biosafety, and navigating regulatory pathways. Future efforts should integrate artificial intelligence-assisted design, multi-stimuli-responsive systems, modular programmable platforms, and physiologically relevant evaluation models such as organoids and microfluidics. With these advances, SAPHs are expected to transition from “proof-of-concept” to “clinical tool”, offering more efficient, safer, and personalized solutions for the precision treatment of mucosa-associated diseases.

## Figures and Tables

**Table 1:** **Comparison of hydrogel types for mucosal drug delivery.**

Hydrogel Type	Building Blocks	Advantages	Disadvantages	Mucosal Applications
SAPHs	Self-assembling peptides	Programmable, biocompatible, in situ gelation, stimuli-responsive	Mechanical strength, batch consistency, cost	Oral, GI, ocular, vaginal, middle ear, nasal
Natural polymer hydrogels	Alginate, chitosan, hyaluronic acid	Biocompatible, abundant, low cost	Batch variability, weak mechanical strength, rapid degradation	Oral, GI, ocular, vaginal
Synthetic polymer hydrogels	PEG, PNIPAM, PLGA-PEG	Tunable mechanical properties, reproducible synthesis	Non-biodegradable residues, low bioactivity	Ocular, vaginal, nasal
Peptide–polymer hybrid hydrogels	Peptide + synthetic/natural polymer	Combines programmability with mechanical robustness	Complex synthesis, regulatory challenges	Ocular, GI, nasal
Hydrogel nano/microcapsules	Peptide or polymer shell	High encapsulation efficiency, controlled release	Limited mucosal retention, complex manufacturing	Oral, GI
Microneedles	Crosslinked peptides/polymers	Painless, bypass mucus barrier	Limited to accessible mucosa, skin irritation	Buccal, ocular, vaginal
Hybrid systems (e.g., liposome-in-hydrogel)	Lipid + hydrogel	Sustained release, enhanced penetration	Stability, scale-up challenges	Ocular, nasal, GI

**Table 2:** **Design principles of self-assembling peptide sequences for mucosal delivery.**

Design Element	Sequence Motif/Example	Function	Mucosal Relevance
β-sheet	(RADA)_4_, (FKFE)_2_	Mechanical strength, hydrogel formation	Resistance to clearance
α-helix	Coiled-coil heptad repeats	Transmembrane penetration	Epithelial barrier crossing
β-hairpin	VPGVG flanked by β-strands	On-demand gelation	In situ retention
Amphiphilic	Hydrophobic tail + hydrophilic head	Nanofiber formation	Drug encapsulation
Aromatic residues	Phe, Tyr, Trp	π–π stacking, mechanical strength	Sustained release
Cationic residues	Lys, Arg	Electrostatic mucoadhesion	Prolonged retention
Anionic residues	Glu, Asp	pH responsiveness	Site-specific release
Enzyme-cleavable	MMP-2 substrates (VSLRA, ASLRA)	Pathological trigger	Inflamed mucosa
CPP	TAT, Penetratin	Transmembrane delivery	Barrier penetration
Non-natural/D-amino acids	D-Phe, D-Ala	Enzymatic stability	Prolonged duration

**Table 3:** **Representative studies of SAPHs in mucosal drug delivery.**

Peptide Sequence	Cargo	Experimental Category	Disease Model	Effect	Limitation	Reference
Carrier: RQDLKTEIRYDFKADE (Rqdl10)	TFF3	In vitro and in vivo	Gastric mucosal injury	1. promoted the repair of gastric mucosal damage; 2. promoted the proliferation and migration of gastric mucosal epithelial cells; 3. inhibited inflammation and apoptotic responses.	Long-term stability and safety unknown; no large animal data	[11]
Carrier: G(RADA)_4_	Sulfasalazine and insulin	In vitro	-	1. pH-responsive drug release; 2. targeted intestinal delivery	No in vivo validation; release kinetics in dynamic GI environment unknown	[16]
Carrier: GFFYD	Chloramphenicol	In vitro and in vivo	Bacterial keratitis	1. antimicrobial; 2. Ca^2+^-regulated elasticity; 3. increased pre-corneal retention	Long-term ocular safety unknown; no clinical data	[84]
AMP domain: AKRHHGYKRKFH; specific targeting domain: TFFRLFNR; enhancing remineralization domain: DSSDSSDSSDSSDSSDSSDSSDSS	Antimicrobial peptide (AMP) (P-113)	In vitro and in vivo	Dental Caries	1. antimicrobial; 2. in situ biomimetic remineralization.	Limited clinical validation; long-term effects on oral microbiome unknown	[104]
Negatively charged peptide amphiphile (PA): C16V2A2E2; positively charged antimicrobial peptide (GL13K): GKIIKLKASLKLL	Antimicrobial peptide (GL13K):	In vitro and in vivo	Periodontitis	1. sustained GL13K release; 2. antibacterial 3. immunomodulatory; 4. inhibition of osteoclastogenesis.	Injection required; long-term effects on periodontal tissue unknown	[108]
Nitroreductase responsiveness: NQ-GGGFF	*Escherichia coli Nissle 1917* (EcN)	In vitro and in vivo	Ulcerative colitis	1. downregulated inflammation; 2. repaired intestinal barrier.	Safety of live cells; long-term colonization effects unknown	[109]
Self-assembled motifs: Nap-FFF	Fibronectin-mimetic peptide (PHSRN)	In vivo	Corneal scrape	1. enhanced the chemical stability; 2. promoted the corneal restoration	Mechanical properties not optimized; no large animal data	[121]
ALP-triggered motifs: Nap-YpYY	TB500 (LKKTETQ)	In vitro and in vivo	Corneal alkali burn	1. ALP-triggered nanofiber formation and gelation in situ; 2. promoted the corneal restoration	Long-term efficacy unknown; no clinical data	[122]
Carrier: YENPTY	Dexamethasone	In vitro and in vivo	Uveitis	1. increased the pre-corneal retention; 2. efficient cellular uptake; 3. suppressed inflammation;	Limited drug loading; long-term safety unknown	[134]
Cell-penetrating peptide 1:KVRVRVRV^D^PPTRVRYRVK, where ^D^P is D-proline; positively charged peptide amphiphile 2:VKVWVKV^D^PPTKVKVKV	Aflibercept	In vitro and in vivo	Corneal neovascularization	1. superior membrane permeability; 2. increased stability; 3. prolonged drug retention time; 4. reduced the density of neovascularization	Safety in humans unknown; cost of biologics	[130]
Carrier: spider silk protein particles	*Periplaneta americana* (PA) extract	In vitro and in vivo	Normal murine	prolonged vaginal retention time	Limited clinical translation; component complexity	[131]
Carrier and permeation enhancer: c16-AHL_3_K_3_	Ciprofloxacin	In vitro	-	1. controlled drug release; 2. the permeation of tympanic membrane	No in vivo data; long-term middle ear safety unknown	[132]

**Table 4:** **Comparison of SAPHs with other mucosal delivery systems.**

System	Advantages	Limitations	Mucosal Applications
SAPHs	Programmable sequence design; excellent biocompatibility; in situ gelation; stimuli-responsive release	Modest mechanical strength; batch-to-batch variability; high cost; regulatory uncertainty	Oral, gastrointestinal, ocular, vaginal, middle ear, nasal
Nanoparticles	High surface area; tunable release; diverse cargo encapsulation	Mucus barrier; rapid clearance; limited penetration	Oral, gastrointestinal, ocular, nasal
Liposomes	Biocompatible; encapsulate hydrophilic and hydrophobic drugs	Physical instability; rapid clearance; scale-up challenges	Ocular, nasal, gastrointestinal
Micelles	Solubilize hydrophobic drugs; small size	Dilution instability; poor mucosal retention	Ocular, oral
Natural polymer hydrogels	Biocompatible; abundant; low cost	Batch variability; weak mechanical strength; rapid degradation	Oral, gastrointestinal, ocular, vaginal
Microneedles	Painless; bypass mucus barrier; precise dosing	Limited to accessible mucosa; manufacturing complexity	Buccal, ocular, vaginal

**Table 5:** **Approved mucosal delivery products and their limitations.**

Product	Route	Active Agent	Clinical Status	Limitations
Nasal spray	Nasal	Calcitonin	Approved	Low bioavailability
Buccal tablet	Buccal	Fentanyl	Approved	Variable absorption
Ocular insert	Ocular	Pilocarpine	Approved	Foreign body sensation
Vaginal ring	Vaginal	Contraceptives	Approved	Local irritation
SAPH-based	Mucosal	—	Preclinical	No approved product

**Table 6:** **Design strategies, release mechanisms, mucosal retention, and penetration enhancement of SAPHs at different mucosal sites.**

Mucosal Site	Key Barrier	Design Strategy	Self-Assembly Trigger	Release Mechanism	Mucosal Retention	Penetration Strategy
Oral cavity	Salivary flushing, lysozyme	Cationic residues, Fmoc modification	pH, ions	Diffusion, enzyme-triggered	Electrostatic + hydrophobic	CPP (TAT, Penetratin)
Stomach	Acid, pepsin	Acid-stable sequence, pH-responsive	pH	pH-triggered disassembly	Bioadhesion	—
Intestine	Mucus, enzymes, pH gradient	pH/enzyme-cleavable	pH, nitroreductase	pH/enzyme-triggered	Mucus interpenetration	CPP, mucolytic agents
Ocular surface	Tear flushing, corneal barrier	Ion-responsive, shear-responsive	Ca^2+^, shear	Diffusion, ion-triggered	Ca^2+^-enhanced elasticity	CPP
Vagina	Mucus flow, microbiota	Thermosensitive composite	Temperature	Sustained diffusion	Mechanical anchoring	—
Middle ear	Tympanic membrane	Dynamic nanopeptide assembly	Concentration-dependent	Diffusion	—	CPP-mediated
Nasal cavity	Mucociliary clearance	Bioadhesive hydrogel	—	Sustained release	Bioadhesion	—

## Data Availability

No new data were created or analyzed in this study. Data sharing is not applicable to this article.
